# A systems biology analysis protein-protein interaction of NASH and IBD based on comprehensive gene information 

**Published:** 2017

**Authors:** Reza Karbalaei, Mehran Piran, Mostafa Rezaei-Tavirani, Hamid Asadzadeh-Aghdaei, Mohammad Hossein Heidari

**Affiliations:** 1 *Proteomics Research Center, Faculty of Paramedical Sciences, Shahid Beheshti University of Medical Sciences, Tehran, Iran*; 2 *Drug Design and Bioinformatics Unit, Medical Biotechnology Department, Biotechnology Research Center, Pasteur Institute of Iran, Tehran, Iran*; 3 *Basic and Molecular Epidemiology of Gastrointestinal Disorders Research Center, Research Institute for Gastroenterology and Liver Diseases Shahid Beheshti University Medical Sciences, Tehran, Iran*

**Keywords:** Inﬂammatory bowel diseases (IBD), Non-alcoholic steatohepatitis (NASH), Protein-protein interaction (PPI) network analysis, Hub-bottlenecks, Protein clusters

## Abstract

**Aim:**

Analysis reconstruction networks from two diseases, IBD and NASH and their relationship, based on systems biology methods.

**Background:**

IBD and NASH are two complex diseases, with progressive prevalence and high cost for countries. There are some reports on co-existence of these two diseases. In addition, they have some similar risk factors such as age, obesity, and insulin resistance. Therefore, systems biology approach can help to discover their relationship.

**Methods:**

DisGeNET and STRING databases were sources of disease genes and constructing networks. Three plugins of Cytoscape software, including ClusterONE, ClueGO and CluePedia, were used to analyze and cluster networks and enrichment of pathways. Based on degree and Betweenness, hubs and bottleneck nodes were defined.

**Results:**

Common genes between IBD and NASH construct a network with 99 nodes. Common genes between IBD and NASH were extracted and imported to STRING database to construct PPI network. The resulting network contained 99 nodes and 333 edges. Five genes were selected as hubs: JAK2, TLR2, TP53, TLR4 and STAT3 and five genes were selected as bottleneck including: JAK2, TP53, AGT, CYP3A4 and TLR4. These genes were hubs in analysis network that was constructed from hubs of NASH and IBD networks.

**Conclusion:**

Systems biology methods, specifically PPI networks, can be useful for analyzing complicated related diseases. Finding Hub and bottleneck proteins should be the goal of drug designing and introducing disease markers.

## Introduction

 Non-alcoholic steatohepatitis (NASH) is a subtype of Non-alcoholic fatty liver disease (NAFLD) which has the potential to progress to cirrhosis, hepatic failure or hepatocellular carcinoma (1-3). This disease is a liver inflammation caused by fat accumulation in the liver. NASH and NAFLD are common diseases in industrial countries (4) and have been reported in Australia, India, Japan, the Middle East, New Zealand, North America, South America, northern Europe, southern Europe and South East Asia (4). In fact nowadays, NASH is the second liver disease in the United States and will be a gold standard for liver transplant in a few years (5). Biopsy of liver is the only way for diagnosing NASH, but it is a costly and error-prone method (6) with some risks (7). So, reducing the cost of early diagnosis of this disease is very important (8).

Inflammatory bowel disease (IBD) is a chronic gastrointestinal disorder that consists primarily of two types: ulcerative colitis and Crohn's disease. Both usually involve severe diarrhea, pain, fatigue and weight loss. This disease is caused by dysregulated immune response to host intestinal microﬂora (9). IBD can be debilitating and sometimes leads to life-threatening complications (10). IBD patients may develop some complicated diseases such as sclerosing cholangitis and autoimmune hepatitis. Most occurrences of IBD are seen in North America, resulting in high costs of health care measures (11). IBD patients are more likely to show overweight and obesity (12-14). Interestingly, these obese persons are at risk of NASH (15). Some scientist report the incidence of NASH in IBD in range of 6.2% to 40% (16-18). Gisbert et al analyzed 786 IBD patients (49% CD and 51% UC subgroup) and reported 40.8% prevalence of NASH in these patients (16). In a study by Sourianarayanane et al, the same investigation was performed in 928 patients (53% CD and 47% UC subgroup) to find an incidence of only 8.2%. Indeed, some NASH risk factors were reported in IBD population, such as small bowel surgery (13), hypertension (13), obesity (13), steroid use (13), active disease (19), duration of IBD (19), prior IBD surgery (19) and anti-TNFα use (19). However, the pathogenesis of NASH in IBD patients is a mystery because of disease-specific risk factors, such as chronic inflammation, drug-induced hepatotoxicity, steroid exposure, malnutrition and gut dysbiosis that is shared between both diseases (20, 21). Systems biology methods can be helpful to provide a new perspective of shared molecular mechanisms in related diseases such as IBD and NASH (22-25). Protein-protein interaction (PPI) network analysis is one of the major fields in systems biology in which analyzed complex interactome of proteins as a main source of data (26). Using systems biology method such as comparison between gene sets of diseases, constructing PPI network and pathway enrichment can be helpful to decipher the shared mechanism of IBD and NASH. In this study, we reported seven important shared proteins between these diseases that can be used not only as markers of disease, but also as targets for drug designing. 

## Methods

DisGeNET is a discovery platform containing one of the largest publicly available collections of genes and variants associated with human diseases (27). The related genes of IBD and NASH were exported from DisGeNET database and used to construct PPI network. The Search Tool for the Retrieval of Interacting Genes/Proteins (STRING), a database for predicted protein-protein interactions at EMBL clusters the extracted results from many protein-protein interactions databases, like Mint, BioGrid, etc. It also uses the information from KEGG pathways and reactome to provide the best annotations for the interactions of one protein (28). We constructed IBD and NASH networks by submitting gene list to STRING database and analyzed the networks by Cytoscape software (29).

A network is composed of nodes (e.g., genes or proteins) and edges/links (e.g., co-expression relationships or physical interactions). In network biology terms, degree, and Betweenness are important centrality parameters that are useful for analysis network topology. Edges/links of a node are called the degree of that node. Nodes with high degree are called hubs and nodes that achieve top-ten or top-five percent of betweenness centrality are called bottlenecks (both based on researcher’s definition) (30). So, nodes that are simultaneously hubs and bottlenecks are named hub-bottlenecks (31). Average degree (A.D) and standard deviation (SD) of degrees were calculated and nodes with degree above two*SD + A.D were selected as hub proteins in each network. Also, the top five percent of betweenness centrality measures were selected as bottleneck proteins. Shared genes, hubs and bottleneck proteins of these two networks were extracted and used for further analysis. The common network was constructed by importing shared genes in STRING database and clustered by ClusterONE plugin of Cytoscape software (32) that finds overlapping protein complexes in a protein interaction network loaded into Cytoscape. (overlap threshold = 1, node penalty = 0, haircut threshold = 0) (33). Pathway enrichment and the relation between pathways were accomplished using ClueGO and CluePedia plugins of Cytoscape software (34, 35). 

**Figure 1:**
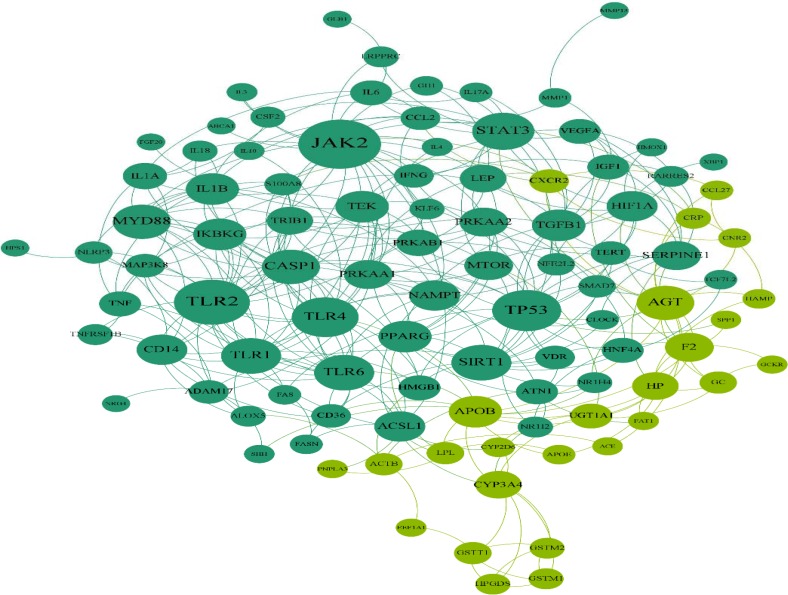
Common gene network containing 99 nodes and 333 edges. This network includes two modules that are highlighted by dark green (cluster one) and light green (cluster two

**Figure 2:**
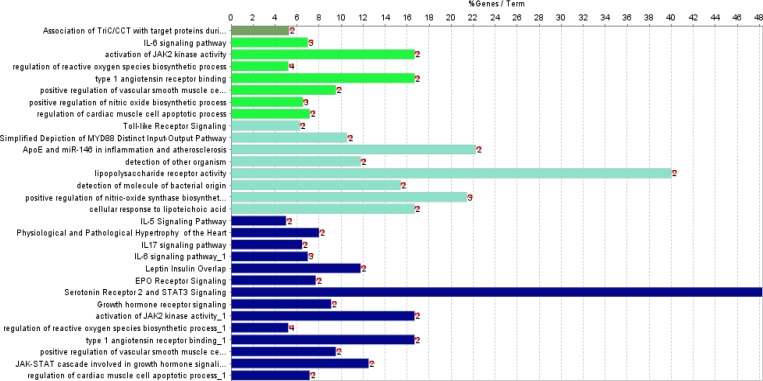
Enriched pathways by seven genes that are shared between table 1 and table 2. Pathways are colored based on classification

**Table 1:** Shared Hub and Bottleneck genes of IBD and NASH networks. Hub-bottleneck genes that are the same as table 1 are marked with asterisks

Hubs	Degree	Bottlenecks	B.N
GMPS	3	SIRT1	0.116186
PRKAG2	12	TP53**	0.100492
MLST8	9	HIF1A	0.086405
MTOR	18		
MAP3K7	22		
TRAF6	19		
PRKAG1	12		
PRKAB1	13		
PRKAB2	12		
ACTB	3		
ACTG1	4		
PRKAA2	14		
SERPINC1	5		
F2	7		
RPTOR	18		
TLR4**	17		
IKBKG	18		
BIRC3	12		
TRAF3	11		
TICAM1	11		
UBE2N	12		
BIRC2	14		
MYD88	15		
IKBKB	21		
SERPIND1	5		
FGG	11		
PRKAA1	16		
TP53**	20		
CREBBP	22		
MDM4	5		
HIF1A	10		
CD14	12		
TLR2**	18		
AGT**	9		
TERT	6		
STAT3**	17		
JAK2**	20		
HMGB1	9		
SIRT1	9		
PPARG	9		
VEGFA	8		
LEP	11		
IL6	3		
IGF1	6		
IL1B	8		
TGFB1	11		
IFNG	4		
SERPINE1	7		
IL4	3		
CSF2	4		
APOB	7		
GH1	3		
TEK	11		
CYP3A4**	2		

**Table 2:** Hub and Bottleneck genes of Common network genes. Hub-bottleneck genes are bolded

Hubs	Degree	Bottlenecks	Betweenness score
JAK2	25	JAK2	0.160941
TLR4	22	TP53	0.10348
TP53	19	AGT	0.102813
TLR2	18	CYP3A4	0.083948
STAT3	16	TLR4	0.083942

**Table 3.** Enriched pathways list, based on seven genes that are shared between table 1 and table 2

GO Term	Ontology Source	Adj-PValue	Associated Genes Found	GO Groups
Association of TriC/CCT with target proteins during biosynthesis	REACTOME	160.0E-6	STAT3, TP53	0
IL-6 signaling pathway	WikiPathways	7.1E-6	AGT, JAK2, STAT3	1
activation of JAK2 kinase activity	GO_BiologicalProcess	100.0E-6	AGT, JAK2	
regulation of reactive oxygen species biosynthetic process	GO_BiologicalProcess	180.0E-9	AGT, JAK2, STAT3, TLR4	
type 1 angiotensin receptor binding	GO_MolecularFunction	100.0E-6	AGT, JAK2	
positive regulation of vascular smooth muscle cell proliferation	GO_BiologicalProcess	210.0E-6	AGT, JAK2	
positive regulation of nitric oxide biosynthetic process	GO_BiologicalProcess	8.3E-6	AGT, JAK2, TLR4	
regulation of cardiac muscle cell apoptotic process	GO_BiologicalProcess	210.0E-6	AGT, JAK2	
Toll-like Receptor Signaling	WikiPathways	170.0E-6	TLR2, TLR4	2
Simplified depiction of MYD88 distinct input-output pathway	WikiPathways	190.0E-6	TLR2, TLR4	
ApoE and miR-146 in inflammation and atherosclerosis	WikiPathways	62.0E-6	TLR2, TLR4	
detection of other organism	GO_BiologicalProcess	170.0E-6	TLR2, TLR4	
lipopolysaccharide receptor activity	GO_MolecularFunction	18.0E-6	TLR2, TLR4	
detection of molecule of bacterial origin	GO_BiologicalProcess	110.0E-6	TLR2, TLR4	
positive regulation of nitric-oxide synthase biosynthetic process	GO_BiologicalProcess	220.0E-9	JAK2, TLR2, TLR4	
cellular response to lipoteichoic acid	GO_BiologicalProcess	100.0E-6	TLR2, TLR4	
IL-5 Signaling Pathway	WikiPathways	90.0E-6	JAK2, STAT3	3
Physiological and Pathological Hypertrophy of the Heart	WikiPathways	240.0E-6	AGT, STAT3	
IL17 signaling pathway	WikiPathways	210.0E-6	JAK2, STAT3	
IL-6 signaling pathway	WikiPathways	7.1E-6	AGT, JAK2, STAT3	
Leptin Insulin Overlap	WikiPathways	170.0E-6	JAK2, STAT3	
EPO Receptor Signaling	WikiPathways	220.0E-6	JAK2, STAT3	
Serotonin Receptor 2 and STAT3 Signaling	WikiPathways	11.0E-6	JAK2, STAT3	
Growth hormone receptor signaling	REACTOME	210.0E-6	JAK2, STAT3	
activation of JAK2 kinase activity	GO_BiologicalProcess	100.0E-6	AGT, JAK2	
regulation of reactive oxygen species biosynthetic process	GO_BiologicalProcess	180.0E-9	AGT, JAK2, STAT3, TLR4	
type 1 angiotensin receptor binding	GO_MolecularFunction	100.0E-6	AGT, JAK2	
positive regulation of vascular smooth muscle cell proliferation	GO_BiologicalProcess	210.0E-6	AGT, JAK2	
JAK-STAT cascade involved in growth hormone signaling pathway	GO_BiologicalProcess	160.0E-6	JAK2, STAT3	
regulation of cardiac muscle cell apoptotic process	GO_BiologicalProcess	210.0E-6	AGT, JAK2	

## Results

**Figure 3:**
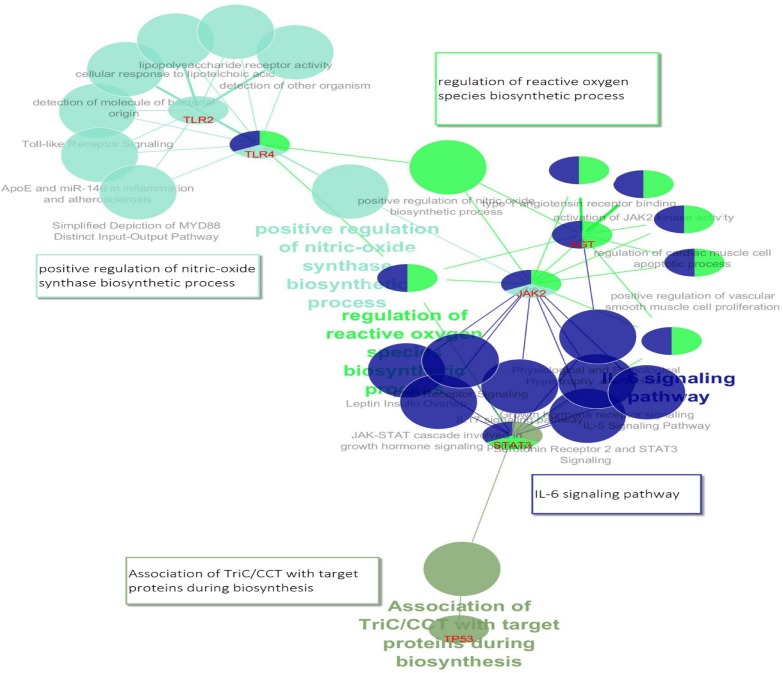
CluePedia map of enriched pathways, main pathways and their relation to genes are shown. Four groups of GO in table 3 are shown in this figure schematically. Group 0: Association of TriC/CCT with target proteins during biosynthesis, dark green; Group 1: IL-6 signaling pathway, dark blue; Group 2: regulation of reactive oxygen species biosynthetic process, light green; positive regulation of vascular smooth muscle cell proliferation, light blue

From DisGeNET, 838 and 331 genes were extracted for IBD and NASH, respectively. Totally, 113 genes were shared between the two lists and were named common genes. The common genes network that was constructed using STRING database, has 99 nodes and 333 edges and two clusters (figure 1). Five genes were selected as hubs: JAK2, TLR2, TP53, TLR4 and STAT3; and five genes were selected as bottleneck including: JAK2, TP53, AGT, CYP3A4 and TLR2 (Table 1). Therefore, in this network, we have three hub- bottleneck genes: JAK2, TLR2 and TP53. Also clustering yielded two cluster: cluster one with 73 nodes (p-value: 0.00004) and cluster two with 23 nodes (p-value: 0.0032) (figure 1). In addition, IBD and NASH networks were constructed by STRING database. The IBD network from STRING analysis consists of 653 nodes and 4110 edges, while the NASH network comprises 257 nodes and 965 edges. Analyzing IBD and NASH networks showed that 54 hubs and three bottlenecks were common between these two networks (Table 2). All hubs and bottlenecks in the common gene network appeared in the list of common hubs from IBD and NASH networks (Table2). So these seven genes were used for pathways enrichment and gene ontology. Gene ontology results showed 24 pathways that can be classified in four clusters (Table 3 and figure 2). These clusters were named based on the main cluster’s pathway: 1. Association of TriC/CCT with target proteins during biosynthesis; 2. IL-6 signaling pathway; 3. Regulation of reactive oxygen species biosynthetic process; and 4. Positive regulation of nitric oxide biosynthetic process. Due to shared mechanism, some pathways appeared in more than one cluster. CluePedia analysis showed that IL-6 signaling pathway group was the central group that connects other groups (Figure3).

## Discussion

Systems biology methods such as PPI network analysis and pathway enrichment have been used broadly to discover main proteins and pathways underlay complex diseases (36). Different types of cancers, various kinds of neurodegenerative diseases and disorders and also many cellular condition are analyzed via protein-protein interaction method (37-42) The co-existence of NAFLD with IBD is becoming increasingly recognized (43). In this study, we used the complete genes list of the two diseases (IBD and NASH) that may have shared mechanism based on risk factors and previous studies (44). According to network analysis of the common network (see table 1) and also findings in table 2, it is indicated that seven key genes are related to the two diseases. The comparative study of several diseases has shown that there are common informatics biomarker panels in the studied cases (38, 39) . Five of the seven selected genes (JAK2, TLR2, TP53, TLR4 and STAT3) were in one cluster in Common genes network. The Janus kinase/signal transducer and activator of transcription (JAK/STAT) signaling cascade is a central pathway whose regulation is important for a variety of biological processes and whose disruption can cause progressive NASH (45). Also, Janus kinase (JAK) inhibitors have been developed as a new small molecule therapy for autoimmune disease such as IBD (46). On the other hand, Interleukin-6 (IL-6) is among the many cytokines that activate JAK/STAT signaling. Knockout of IL-6 gene affects mice with obesity and NASH (47). Interestingly, the role of IL-6 in IBD immunopathogenesis and its clinical relevance in IBD therapy and diagnostics are well studied (48). Also, TLR2 and TP53 have important roles in both IBD and NASH. TLRs are key mediators of innate host defense in the intestine, involved in maintaining mucosal as well as commensal homeostasis (49). Findings in diverse murine models of colitis have revealed the vital role of TLR dysfunction in IBD pathogenesis (50). Cengiz et al showed that serum TLR4 levels were elevated in NASH patients in comparison with healthy controls. Moreover, in NASH patients, serum level of TLR4 was able to predict liver fibrosis (51). TP53, encoding p53 protein, triggers apoptosis in NASH (52) and IBD patients (53). The finding (see table 3) indicates that JAK2 is involved in 20 biological processes among 30 GO terms (about 70% attributions). STAT3 and AGT, the other two genes, are related to about 40% of terms and TP53 the common genes in many cancer diseases is involved only in one term. Involvement of the key genes in the cluster of biological processes is shown in the figure 3. As depicted in this figure, the central role of TLR4, STAT3 and JAK2 is highlighted. It seems that JAK2, STAT3 and AGT are the most important genes that are connected closely to the two compared diseases.

These results showed that application of systems biology methods unravels the secret behind common mechanism of IBD and NASH diseases. The real impact of IBD therapies on co-existing NAFLD also needs to be further assessed. Also, appropriate screening tools and strategies for the management of co-existing diseases in IBD patients are lacking. Clarification of these issues may enhance early intervention and improve patient outcomes.

## Conflict of interests

 The authors declare that they have no conflict of interest.
